# Risk Profiles of Poor Diet Quality Among University Students: A Multivariate Segmentation Analysis

**DOI:** 10.3390/nu17233639

**Published:** 2025-11-21

**Authors:** Luis Moral-Moreno, Elizabeth Flores-Ferro, Fernando Maureira Cid, Ivonne Vizcarra, Alejandra D. Benítez-Arciniega, Edna Graciela García, Manuel E. Cortés

**Affiliations:** 1 Departamento de Didáctica de la Expresión Musical, Plástica y Corporal, Centro de Enseñanza Superior Don Bosco, María Auxiliadora, 9, 28040 Madrid, Spain; 2 Escuela de Ciencias y Tecnología Educativa, Facultad de Educación, Universidad Católica Silva Henríquez, General Jofré, 462, Santiago 8330225, Chile; prof.elizabeth.flores@gmail.com; 3 Departamento de Educación Física, Deportes y Recreación, Universidad Metropolitana de Ciencias de la Educación, Av. José Pedro Alessandri, 774, Ñuñoa, Santiago 7760197, Chile; maureirafernando@yahoo.es; 4 Instituto de Ciencias Agropecuarias y Rurales (ICAR), Universidad Autónoma del Estado de México, Instituto Literario 100 Col. Centro, Toluca 50000, Mexico; ivizcarrab@uaemex.mx (I.V.); abeniteza@uaemex.mx (A.D.B.-A.); ednaegga@gmail.com (E.G.G.); 5 Dirección de Investigación y Programa de Doctorado en Educación, Universidad Bernardo O’Higgins, General Gana, 1670, Santiago 8370993, Chile; cortesmanuel@docente.ubo.cl

**Keywords:** university health, diet quality, dietary behaviors, lifestyle factors, post-pandemic nutrition, predictive analytics

## Abstract

Background: University students often display unhealthy eating habits shaped by economic, cultural, and psychosocial factors. These behaviors increase risks of chronic and mental disorders. The COVID-19 pandemic further changed their diet and physical activity (PA) habits, highlighting the need to identify determinants of diet quality (DQ). Objective: The objective of this study is to identify risk profiles of poor DQ among university students from Chile, Mexico, Spain, and Italy through multivariate segmentation analysis. Methods: A cross-sectional predictive study was conducted among 686 university students (60.8% women; mean age = 22.4 ± 5.1 years) using an online questionnaire on sociodemographic, academic, health, and lifestyle factors, including PA (IPAQ-SF^®^) and DQ (HEI). Analyses included descriptive, inferential, and decision tree (CHAID and CART) models. Results: Significant differences in HEI scores (*p* < 0.001) were observed by country, field of study, academic year, and PA level. Chilean and Mexican students had the lowest DQ. Both models achieved high overall accuracy (≈91%), but balanced accuracy was around 50%, reflecting limited discrimination of healthy diet profiles and underscoring their exploratory value for identifying at-risk subgroups rather than precise prediction. CART identified country of residence and socioeconomic status as the primary determinants of poor diet quality (DQ), while CHAID highlighted field of study and socioeconomic status, with PA and BMI contributing at secondary levels. Conclusions: The results emphasize adapting public health strategies to local contexts—promoting Mediterranean-style diets in European universities and improving access to affordable healthy foods in Latin American campuses, complemented by campus initiatives integrating nutrition education, physical activity, and psychosocial support.

## 1. Introduction

Diet quality (DQ) is a multidimensional construct reflecting not only nutrient adequacy but also adherence to healthy dietary behaviors and balance in food-group consumption. A high-quality diet—rich in fruits, vegetables, whole grains, legumes, and unsaturated fats—supports physiological, metabolic, and psychological well-being, whereas a low-quality diet—characterized by frequent consumption of ultra-processed foods, refined sugars, and saturated fats—has been recognized as one of the leading modifiable risk factors for morbidity and premature mortality worldwide [1,2]. Such dietary behaviors not only compromise individual health but also impose substantial social and economic burdens, underscoring the urgency of early, context-specific preventive strategies.

Cardiovascular health offers a clear example: several studies show a two-way relationship between diet quality and early vascular function in young adults. Diets high in saturated fats, refined carbohydrates, and ultra-processed foods are consistently associated with elevated blood pressure, higher LDL cholesterol, increased triglycerides, and greater visceral adiposity—an adverse constellation that accelerates cardiometabolic risk even before mid-adulthood [2,3]. By contrast, nutrient-dense dietary profiles rich in fruits, vegetables, whole grains, legumes, and unsaturated fats are inversely related to markers of metabolic syndrome and atherogenesis, underscoring diet’s preventative capacity during the university years [4]. In student populations, these associations are not merely theoretical: modest rises in systolic blood pressure or LDL concentrations have been linked to subclinical vascular dysfunction and a higher lifetime risk of coronary artery disease, suggesting that small deteriorations in diet may have outsized long-term consequences [5,6]. Moreover, clustering of modifiable behaviors—physical inactivity, high-fat diets, prolonged sitting—creates synergistic effects that magnify cardiometabolic burden beyond the sum of individual risks [4,6]. These observations point to determinants that extend beyond individual choice to include food environments and socioeconomic constraints shaping access to healthy options. Promoting adherence to Mediterranean or DASH-type habits therefore represents a practical, evidence-based strategy to mitigate early vascular damage and support cardiovascular resilience in university settings.

Diet quality is also intertwined with mental health and disordered eating. Unhealthy eating behaviors commonly co-occur with body-image concerns and dysregulated habits—restrictive intake alternating with binge episodes or compensatory practices—heightening risk for binge-eating disorder, bulimia nervosa, or night-eating syndrome during the transition to university life, when academic pressure and social comparison often intensify [7,8]. Beyond clinical syndromes, dietary behaviors exert a broader influence on psychological wellbeing. Diets deficient in omega-3 fatty acids, vitamins, and antioxidants—and enriched in ultra-processed products—have been associated with greater depressive and anxiety symptoms, chronic stress, poorer sleep, and diminished cognitive performance and academic achievement [8,9,10]. Conversely, meta-analytic evidence indicates that healthier profiles, including Mediterranean and plant-forward diets, are linked to lower incidence of depressive symptoms and improved emotional regulation [11,12,13]. Biologically, energy-dense, nutrient-poor diets may contribute to systemic inflammation, gut dysbiosis, and neurochemical dysregulation, pathways plausibly exacerbating mood disturbances [10,14,15]. Overall, the evidence suggests that nutrition influences not only physical health but also psychological well-being, highlighting its relevance in university settings.

University students represent a critical population for studying diet-related behaviors. The transition from adolescence to adulthood coincides with greater autonomy in food choices, exposure to new social environments, and academic demands that disrupt established routines [4,16]. These changes are related to irregular meal timing, skipping breakfast, increased consumption of snacks and fast food, and reduced intake of fresh produce [17,18]. Recent multicenter studies reveal that fewer than one in three university students meet WHO recommendations for daily fruit and vegetable intake, while consumption of sugary beverages, processed meats, and energy-dense snacks remains alarmingly high [2,4,18]. Environmental factors also play a significant role: university canteens and vending facilities frequently offer inexpensive but low-nutrient options, and the prevalence of sedentary lifestyles further compounds unhealthy habits [13]. The post-pandemic period has intensified these trends, as remote learning, limited socialization, and altered physical activity routines have reshaped daily habits and increased psychosocial stress [13,16].

Within this context, understanding the determinants of diet quality in university students is crucial for designing effective public health interventions. Prior research has identified multiple correlates, including sociodemographic factors such as gender, age, and place of origin [16,18,19], socio-economic indicators like income and parental education [20,21,22], academic factors such as field of study or year of enrollment [4,18], and behavioral dimensions such as physical activity, sleep, and BMI [23,24,25]. Evidence shows that students with higher socioeconomic status or those who regularly engage in physical activity generally report healthier dietary habits, whereas financial strain, independent living, and extended study hours are associated with poorer diet quality [4,16,26]. These factors interact in complex ways that are not easily explained by traditional linear analyses. Recent studies have therefore applied multivariate, data-driven approaches—such as classification and regression trees or machine-learning models—to more accurately describe these interactions [25].

Among these, Classification and Regression Trees (CART) and Chi-squared Automatic Interaction Detector (CHAID) stand out as flexible and interpretable algorithms capable of identifying patterns of interaction between multiple predictors and outcomes [27]. Unlike regression-based methods, these decision-tree models are non-parametric, do not assume linearity, and can simultaneously handle both categorical and continuous variables [28]. By segmenting the population into homogeneous subgroups, they reveal how combinations of variables—rather than isolated factors—define specific risk profiles. CART prioritizes model simplicity and predictive accuracy through pruning and cross-validation, whereas CHAID emphasizes statistically significant multiway splits based on chi-square tests, generating a hierarchical and easily interpretable structure [27]. In this field of study, the combined application of CHAID and CART provides a powerful and complementary framework for understanding how multiple determinants converge to shape dietary behaviors among university students. Together, these decision-tree algorithms generate interpretable rules that uncover complex interactions among sociodemographic, academic, and lifestyle variables underlying distinct dietary risk profiles, thereby enhancing the explanatory depth of behavioral segmentation [17,23]. Combining CHAID’s multiway segmentation with CART’s predictive precision made it possible to identify how several health-related behaviors interact across the student population [27,28].

Despite the growing application of advanced analytical methods, few studies have used them to assess diet quality among university students, particularly in cross-national contexts [29]. Most available evidence derives from single-country studies or relies on isolated indicators of dietary behavior, without accounting for the combined influence of socioeconomic and lifestyle variables [30]. Recent research highlights that COVID-19 lockdowns significantly altered students’ eating habits, often leading to increased snacking, higher intake of ultra-processed foods, and decreased consumption of fresh produce [29,30]. However, comparative evidence across cultural and institutional contexts remains scarce. Furthermore, much of the existing literature predates or only partially captures the post-pandemic transition, during which remote learning, restricted mobility, and psychosocial stress reshaped dietary behaviors and mental well-being [30,31]. Overcoming these gaps is essential to design nutrition and health-promotion strategies that truly reflect the daily realities of students in different university contexts.

The present study was designed to respond to these limitations by integrating a multidimensional, data-driven perspective. We aimed to identify cross-national risk profiles of poor diet quality among university students from Chile, Mexico, Spain, and Italy during the post-pandemic period. Using CHAID and CART algorithms, we examined how demographic, socioeconomic, academic, and behavioral factors interact to predict DQ and compared their predictive contributions across countries. We hypothesized that students with lower socioeconomic status, reduced physical activity levels, and higher BMI would show poorer DQ, whereas those living with family members and demonstrating greater health awareness would report higher diet quality. Furthermore, we anticipated that these associations would differ among countries, reflecting cultural and contextual variations in food availability, institutional environments, and social norms.

By identifying the variables and interactions that most strongly predict poor DQ, this research seeks to provide a robust empirical foundation for targeted health-promotion initiatives aligned with the Health-Promoting University framework [32]. This model advocates for universities as settings that integrate education, policy, and environmental change to foster holistic student well-being. Understanding the multifactorial predictors of DQ in this population may thus inform culturally sensitive interventions that promote healthy eating, physical activity, and psychological balance, ultimately supporting students’ comprehensive human development in a post-pandemic world.

## 2. Materials and Methods

### 2.1. Study Design and Participants

This study employed a cross-sectional and analytical design involving university students from four countries representing diverse sociocultural and dietary contexts.

A total of 686 valid responses were retained for the final analysis: 161 from Chile (23.5%), 162 from Mexico (23.6%), 180 from Spain (26.2%), and 183 from Italy (26.7%). Inclusion criteria comprised the following: (i) being an undergraduate student enrolled in a higher education institution, (ii) age ≥ 18 years, and (iii) provision of informed consent. Exclusion criteria included incomplete questionnaires or inconsistent data in key variables (e.g., implausible anthropometric or dietetic values). The final sample consisted of 417 women (60.8%) and 269 men (39.2%), with a mean age of 22.4 ± 5.1 years (range 18–64).

### 2.2. Instruments and Measures

Data were collected using a structured questionnaire administered online through Google Forms^®^. The instrument was originally created in Spanish and subsequently adapted linguistically and culturally for each country using forward–backward translation to ensure semantic and conceptual equivalence. Validation was carried out by national research teams with expertise in nutrition and public health, who reviewed the wording of dietary items and ensured comparability across contexts.

The questionnaire included the following sections:

- Sociodemographic and health variables (sex, age, weight, height, academic program, year of study, self-reported socioeconomic status [SES], cohabitation, employment and financial status, medical treatment or prescribed diet, history of COVID-19 infection, and restrictions on physical activity [PA] during lockdown). Body mass index (BMI, kg·m^−2^) was calculated from self-reported weight and height and subsequently categorized according to WHO criteria [33].
- Diet quality: assessed using the Healthy Eating Index adapted for the Spanish population [34], which evaluates adherence to healthy eating recommendations across nine food components, including overall dietary variety. Each item is scored from 0 to 10, with a total possible score of 100. Based on conventional cut-offs, scores were classified as unhealthy diet (<50), diet needing modification (50–80), or healthy diet (>80). Previous research has demonstrated that the HEI is a valid and reliable indicator of diet quality among university student populations [35,36].
- Physical activity: measured using the International Physical Activity Questionnaire–Short Form (IPAQ-SF^®^) [37]. This seven-item self-report instrument has demonstrated acceptable validity and reliability in student populations in Spain [38] and Chile [39] and is widely used in global surveillance studies [40]. According to WHO guidelines for adults aged 18–64 years [41], respondents were categorized into low, moderate, or high PA levels.

The inclusion of sociodemographic, socioeconomic, behavioral, and anthropometric factors was both theoretically and empirically supported by previous research identifying them as key determinants of diet quality among university students [6,18,19,21,42]. Gender, socioeconomic status, living arrangements, physical activity, and BMI have consistently been linked to adherence to healthy dietary habits in young adult populations [3,13,18,19,42]. Incorporating these predictors enabled a multidimensional assessment of diet quality, capturing the complex interactions between lifestyle, social context, and individual characteristics.

### 2.3. Procedures

Data were collected between November 2023 and April 2024 through an international collaboration among participating partner institutions. A non-probability convenience sampling approach was applied, ensuring voluntary participation without monetary or academic incentives. The survey was administered online via Google Forms under secure institutional accounts. Each university disseminated the link through official channels—such as institutional mailing lists, classroom announcements, and learning platforms—with support from faculty members who facilitated participant outreach and standardized study instructions.

Participants completed the online survey during scheduled class time or remotely, depending on institutional arrangements. The average completion time was approximately 12 min. Before administration, trained faculty provided a standardized explanation of the study objectives and instructions. Responses were anonymous and stored securely.

The online format enabled standardized and reliable data collection. Participants were able to review their responses before submission, and duplicate entries (identified by identical timestamps or IP addresses) were automatically excluded. The final dataset was subsequently screened for missing or inconsistent values, which represented less than 2% of the total sample and were managed using pairwise deletion in the descriptive analyses.

### 2.4. Ethical Considerations

All procedures conformed to the ethical principles of the Declaration of Helsinki (revised 2013). Ethical approval for the study protocol was obtained from the Ethics Committee of the Universidad Autónoma del Estado de México (ICAR code 01-SEP-2023). Informed consent was obtained electronically after students were fully informed of their rights and the conditions of participation.

### 2.5. Statistical Analysis

All analyses were performed using IBM SPSS^®^ Statistics v29.0 (IBM Corp., Armonk, NY, USA). The significance threshold was set at *p* < 0.05 (two-tailed).

Continuous variables were summarized as means, standard deviations, and ranges, while categorical variables were presented as frequencies and percentages. Normality was assessed using the Shapiro–Wilk test and homogeneity of variance with Levene’s test.

Preliminary comparisons were conducted between countries and gender subgroups to describe the sample characteristics. For categorical variables (e.g., food-group consumption, diet quality categories, sociodemographic characteristics), Pearson’s chi-square test (χ^2^) was used, and results were reported with χ^2^ values, degrees of freedom, *p*-values, and effect sizes (Cramer’s V). Adjusted standardized residuals were calculated to identify cells contributing significantly to the overall χ^2^.

Parametric tests were applied only when assumptions of normality and homoscedasticity were satisfied; otherwise, non-parametric alternatives were used. Differences in mean HEI scores between groups were analyzed using one-way ANOVA, followed by Tukey’s or Bonferroni’s post hoc tests when appropriate. Effect sizes were reported as omega squared (ω^2^). The Kruskal–Wallis H test was used to examine HEI differences across ordinal variables, with Dunn’s pairwise comparisons and Bonferroni adjustment for multiple testing. Effect sizes were reported as epsilon squared (ƐR^2^).

Regarding the multivariate segmentation analyses, all predictors described in the theoretical framework were included in the CHAID and CART models to identify combinations of variables that best predicted poor diet quality across countries.

CHAID (Chi-squared Automatic Interaction Detector) and CART (Classification and Regression Tree) were selected because they are non-parametric, data-driven algorithms capable of handling both categorical and continuous predictors, detecting non-linear interactions, and uncovering hierarchical relationships among variables [27,28]. Their joint use enhances model robustness: CHAID emphasizes statistical significance in multiway splits based on chi-square tests with Bonferroni adjustment, whereas CART applies binary recursive partitioning guided by the Gini index and pruning to maximize prediction accuracy.

Using both approaches allowed the identification of stable and interpretable predictors of poor diet quality, providing complementary insights into cross-national risk profiles. Similar applications of these algorithms have been reported in nutritional epidemiology to model complex behavioral determinants [25].

Independent variables entered into the models included sociodemographic (country, gender, socioeconomic status, academic program, year of study, cohabitation, employment, financial situation), health-related (BMI, medical treatment, prescribed diet, COVID-19 infection, PA restrictions), and lifestyle factors (habitual PA level, pre-/post-pandemic PA change, compliance with WHO PA recommendations). For classification trees, HEI was dichotomized as healthy (>80) vs. poor (≤80) DQ, grouping ‘unhealthy’ and ‘needs modification’ into the poor DQ category.

The selection of tree parameters was guided by both theoretical and empirical considerations to balance model interpretability and predictive accuracy. Minimum parent and child node sizes (50 and 25 cases, respectively) were chosen to ensure adequate sample representation within each split and to prevent overfitting in small subgroups, a criterion frequently applied in behavioral and nutritional epidemiology studies [23,27,28]. The maximum tree depth (set at 5) was determined to preserve model parsimony while allowing sufficient complexity to capture relevant interactions among predictors. Ten-fold cross-validation was used to estimate misclassification risk and assess model stability across subsamples.

Model performance was evaluated through risk estimates, confusion matrices, balanced accuracy—which accounts for class imbalance between healthy and poor diet quality categories—and visual inspection of the decision tree to verify the interpretability and coherence of the segmentation pathways. Variable importance scores derived from impurity reduction (Gini index for CART) and chi-square statistics (for CHAID) were used to identify the most influential predictors of diet quality.

Although the term “patterns” is occasionally used in this manuscript to denote statistical groupings of behaviors, the study did not analyze dietary patterns in the nutritional sense (i.e., portion sizes, meal timing, or macronutrient distribution). As the questionnaire captured only the frequency of food-group consumption, the analysis focused on diet quality profiles derived from Healthy Eating Index (HEI) scores. Accordingly, any reference to “patterns” should be understood as referring to behavioral profiles identified through segmentation models rather than to complete dietary pattern characterization.

## 3. Results

### 3.1. Sociodemographic Sample Characteristics

Table 1 summarizes the sociodemographic characteristics of the 686 university students included in the study, distributed across Chile (n = 161), Spain (n = 180), Italy (n = 183), and Mexico (n = 162). Overall, 60.8% were female, although gender distribution differed significantly by country (χ^2^(3) = 64.29, *p* < 0.001; V = 0.31). Post hoc comparisons showed that males were overrepresented in Chile and underrepresented in Italy and Mexico, while females were more frequent in Italy and Mexico.

Field of study displayed the strongest cross-national divergence (χ^2^(21) = 1735.50, *p* < 0.001; V = 0.92). Chilean students were exclusively enrolled in Physical Education Pedagogy; Spanish students mainly in Primary and Early Childhood Education; Italians concentrated in Clinical and Educational Psychology; and Mexicans in double-degree and Educational Therapy. Academic year also differed significantly (χ^2^(12) = 261.04, *p* < 0.001; V = 0.36), with first-year students overrepresented in Chile and fifth-year students in Italy.

Living arrangements varied across countries (χ^2^(3) = 37.44, *p* < 0.001; V = 0.23). Students in Spain and Mexico more often lived with close family, while Italian students more frequently lived with non-family members. Household size also differed (χ^2^(12) = 53.84, *p* < 0.001; V = 0.16), with four-person households more common in Spain and larger families (≥5 members) typical in Mexico.

Socioeconomic status differed markedly (χ^2^(15) = 300.16, *p* < 0.001; V = 0.38). Mexican students were predominantly higher-middle, Chileans lower-middle or low, and Spanish and Italian students mainly middle class. Employment status also varied (χ^2^(3) = 40.48, *p* < 0.001; V = 0.24): employment was highest in Spain and lowest in Mexico. Financial autonomy differed accordingly (χ^2^(9) = 21.38, *p* = 0.011; V = 0.10), with Mexican students more often fully dependent, Spanish students more frequently partially independent (<50% of expenses), and Chileans slightly overrepresented among those covering >50%.

Health-related variables also showed significant contrasts. Current treatment status differed by country (χ^2^(6) = 38.96, *p* < 0.001; V = 0.17): Italians more often reported psychological treatment, Chileans medical treatment, and Mexicans no treatment. Following a medically prescribed diet was more common in Italy and less so in Mexico (χ^2^(3) = 10.51, *p* = 0.015; V = 0.12).

COVID-19-related responses also varied significantly. Students from Chile and Mexico were more likely to report no prior infection, while Spanish participants more often cited self-administered test positives and Italians physician-confirmed cases (χ^2^(6) = 67.95, *p* < 0.001; V = 0.22). Restrictions on physical activity during lockdown differed (χ^2^(9) = 122.42, *p* < 0.001; V = 0.24): non-infection causes were more frequent in Chile and Spain, infection-related rest in Italy, and space-related limitations in Mexico.

Post-pandemic physical activity trends also showed national variability (χ^2^(12) = 79.07, *p* < 0.001; V = 0.20). Chilean students most frequently reported higher activity levels, while Italian students more frequently reported no substantial change in activity. Spanish and Mexican participants reported more declines (z = −1.9 to −3.0). Likewise, compliance with WHO activity recommendations differed (χ^2^(6) = 62.29, *p* < 0.001; V = 0.21): high compliance was greatest in Chile, moderate in Mexico, and low in Italy.

Finally, BMI-based weight status varied significantly across countries (χ^2^(9) = 35.64, *p* < 0.001; V = 0.13). Underweight was more frequent among Italian students, normal weight among Spanish, and overweight and obesity among Mexican participants. These results collectively demonstrate substantial cross-national diversity in students’ sociodemographic, behavioral, and health-related profiles, reflecting cultural, structural, and contextual differences in their living and study environments.

Significant cross-country differences were observed in age (H(3) = 180.60, *p* < 0.001, ƐR^2^ = 0.26), weekly working hours (H(3) = 175.45, *p* < 0.001, ƐR^2^ = 0.48), and indicators of physical activity—vigorous (H(3) = 32.41, *p* < 0.001, ƐR^2^ = 0.05), moderate (H(3) = 32.75, *p* < 0.001, ƐR^2^ = 0.05), walking (H(3) = 11.89, *p* = 0.008, ƐR^2^ = 0.02), and total METs (H(3) = 28.18, *p* < 0.001, ƐR^2^ = 0.04)—as well as in body mass index (BMI) (H(3) = 38.83, *p* < 0.001, ƐR^2^ = 0.06). Italian students were the oldest (mean 26.3 y), while Chilean, Spanish, and Mexican participants clustered around 21 y. Weekly work hours were highest in Chile, Italy, and Spain, and lowest in Mexico. Although Chilean students reported the greatest vigorous and total METs, BMI was highest in Mexican students, followed by Chile, with Spain and Italy presenting lower values (Table 2).

### 3.2. Frequency of Food Groups Consumption by Country

Table 3 shows the frequency of food consumption by food groups for the total sample of students and according to country of residence.

Analysis of food consumption by country (Table 3) revealed significant cross-national differences across most food groups. Chi-square tests showed associations for cereals (χ^2^(12) = 41.91, *p* < 0.001; V = 0.14), vegetables (χ^2^(12) = 76.90, *p* < 0.001; V = 0.19), fruits (χ^2^(12) = 61.46, *p* < 0.001; V = 0.17), dairy (χ^2^(12) = 125.28, *p* < 0.001; V = 0.25), meat/fish/eggs (χ^2^(12) = 44.61, *p* < 0.001; V = 0.15), legumes (χ^2^(12) = 126.51, *p* < 0.001; V = 0.25), processed meats (χ^2^(12) = 115.06, *p* < 0.001; V = 0.24), sweets and baked goods (χ^2^(12) = 80.68, *p* < 0.001; V = 0.20), and sugar-sweetened beverages (χ^2^(12) = 178.89, *p* < 0.001; V = 0.29), with small-to-moderate effect sizes depending on the group.

Descriptively, Italian students reported higher daily consumption of cereals, vegetables, and fruits, along with elevated intake of sweets and baked goods. Spanish students showed the highest consumption of dairy, meat, fish, eggs, and processed meats, while Mexican students led in legume intake. Chilean students exhibited the greatest daily consumption of sugar-sweetened beverages.

Adjusted residual analyses confirmed these country-specific behaviors: Italy showed more daily consumers of cereals and vegetables; Spain and Italy of fruits and dairy; Mexico of legumes; and Chile of sugary drinks. Conversely, Spain reported fewer sweets and baked goods than Mexico and Chile. These patterns highlight distinct national dietary profiles, reflecting cultural and nutritional habits that vary across Mediterranean and Latin American contexts.

### 3.3. Diet Quality

Table 4 shows the prevalence of dietary habits across categorical variables with significant differences. Diet quality varied significantly by country (χ^2^(6) = 26.37, *p* < 0.001; V = 0.14) and field of study (χ^2^(14) = 28.61, *p* = 0.012; V = 0.14), both reflecting moderate associations. Smaller yet significant effects were observed for academic year (χ^2^(8) = 16.43, *p* = 0.037; V = 0.11) and habitual physical activity level (χ^2^(4) = 13.02, *p* = 0.011; V = 0.10), while no other categorical variables showed significant associations (*p* ≥ 0.05).

Adjusted standardized residuals revealed that Italian students had the highest proportion of healthy diet group (HEI > 80), whereas Mexican and Chilean students were overrepresented in the poor diet quality groups (HEI ≤ 80). Physical Education Pedagogy students were more likely to report lower diet quality, while Clinical and Educational Psychology students tended toward healthier habits. Fourth-year students were most represented in the healthy diet group, in contrast with fifth-year students, and those reporting low habitual physical activity were more often classified within the poor-quality diet categories.

Across countries, no significant associations were observed between diet quality and either field or year of study, suggesting consistent dietary patterns across academic disciplines and university stages. Conversely, habitual physical activity was significantly related to diet quality in Italy (χ^2^(4) = 9.98, *p* = 0.041; V = 0.17) and Mexico (χ^2^(4) = 13.52, *p* = 0.009; V = 0.20), with more active students more frequently classified within the healthy diet category. Although over 20% of the expected counts were below five—warranting cautious interpretation—these results indicate general homogeneity across academic factors and a context-dependent link between physical activity and healthier eating habits.

Table 5 presents HEI scores for variables showing significant differences. ANOVA revealed country-level effects (F(3, 682) = 17.53, *p* < 0.001, ω^2^ = 0.07, 95% CI = 0.032–0.104): Mexican and Chilean students scored lower than Spanish and Italian peers (*p* < 0.001), with no difference between Spain and Italy (*p* = 0.95). Field of study also varied significantly (F(7, 678) = 7.01, *p* < 0.001, ω^2^ = 0.06), with lower HEI scores among Education and Educational Therapy students compared with Primary and Educational Psychology (*p* ≤ 0.01). When analyzed by country, the association between study program and diet quality was nonsignificant in Chile, Spain, and Mexico, but marginal in Italy (H(7) = 14.72, *p* = 0.040, r = 0.18), where Educational Psychology and Primary Education students scored higher.

Academic year was associated with HEI (F(4, 681) = 5.89, *p* < 0.001, ω^2^ = 0.03): first- and third-year students scored lower than second- and fourth-year peers. Only Mexico showed a country-specific effect (F(4, 157) = 2.46, *p* = 0.048, ω^2^ = 0.06), with second-year students outperforming third-year peers.

HEI scores also differed by habitual physical activity (F(2, 698) = 4.03, *p* = 0.018, ω^2^ = 0.01), with high-activity students scoring higher than those with low activity (*p* = 0.015). This relationship was significant only in Italy (F(2, 180) = 3.73, *p* = 0.026, ω^2^ = 0.04), where more active students reported healthier diets.

BMI-based weight status was associated with HEI (F(3, 682) = 3.06, *p* = 0.028, ω^2^ = 0.01); normal-weight and overweight students scored higher than obese peers (*p* ≤ 0.042). No within-country differences emerged, though obese students consistently displayed slightly lower HEI means.

Socioeconomic status also influenced HEI (H(4) = 19.85, *p* < 0.001, ƐR^2^ = 0.03), with higher SES groups scoring above all others. However, when analyzed separately by country, socioeconomic status showed no significant association with HEI (*p* ≥ 0.05), suggesting that diet quality remained relatively consistent across socioeconomic strata.

Household size showed a weak association (H(4) = 9.77, *p* = 0.044, ƐR^2^ = 0.01): students living alone scored slightly lower than those living with multiple cohabitants. However, when analyzed by country, this association reached significance only in Italy (H(4) = 13.69, *p* = 0.008, ƐR^2^ = 0.05), suggesting that living arrangements influenced diet quality mainly in that context.

Finally, pre/post-pandemic physical activity change correlated with HEI (H(4) = 19.14, *p* < 0.001, ƐR^2^ = 0.03), with students reporting increased activity showing higher diet quality. When stratified by country, this association remained significant in Italy (H(4) = 9.98, *p* = 0.041, ƐR^2^ = 0.03) and Mexico (H(4) = 13.52, *p* = 0.009, ƐR^2^ = 0.04), suggesting that the positive link between active lifestyles and healthy eating was most evident in these contexts.

### 3.4. Predictors of Poor Diet Quality

#### 3.4.1. Exhaustive CHAID Model

**Figure 1:**
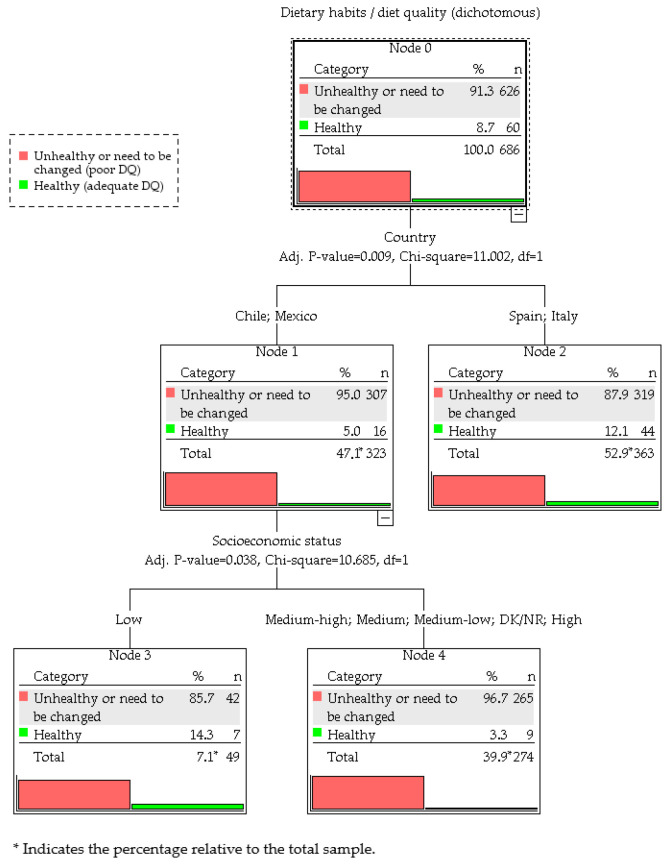
Exhaustive CHAID classification tree for diet quality.

The exhaustive CHAID analysis produced a three-terminal-node classification structure based on two predictors (Figure 1). Country of residence was the most discriminating variable (adjusted *p* = 0.009; χ^2^ = 11.00; *df* = 1). Students from Chile and Mexico (Node 1) had a higher prevalence of poor-quality diet (HEI ≤ 80) (95.0%) compared with Spain and Italy (Node 2; 87.9%).

Within the Latin American subgroup (Node 1), SES emerged as a second predictor (adjusted *p* = 0.038; χ^2^ = 10.68; *df* = 1). Students reporting low SES (Node 3) had a lower prevalence of poor diet quality (85.7%) than peers with medium to high SES (Node 4; 96.7%).

Model performance showed Node 4 (42.3% of poor DQ cases), Node 2 (51.0%), and Node 3 (6.7%) as the most influential segments.

The Exhaustive CHAID model yielded an overall accuracy of 91.3% (risk estimate = 0.087, SE = 0.011); however, this value was driven entirely by the majority “poor diet quality” class (91.2% of cases). The model correctly classified all students with poor DQ (sensitivity = 100%) but failed to identify any students with healthy DQ (specificity = 0%), resulting in a balanced accuracy of 50%.

#### 3.4.2. CART Model

**Figure 2:**
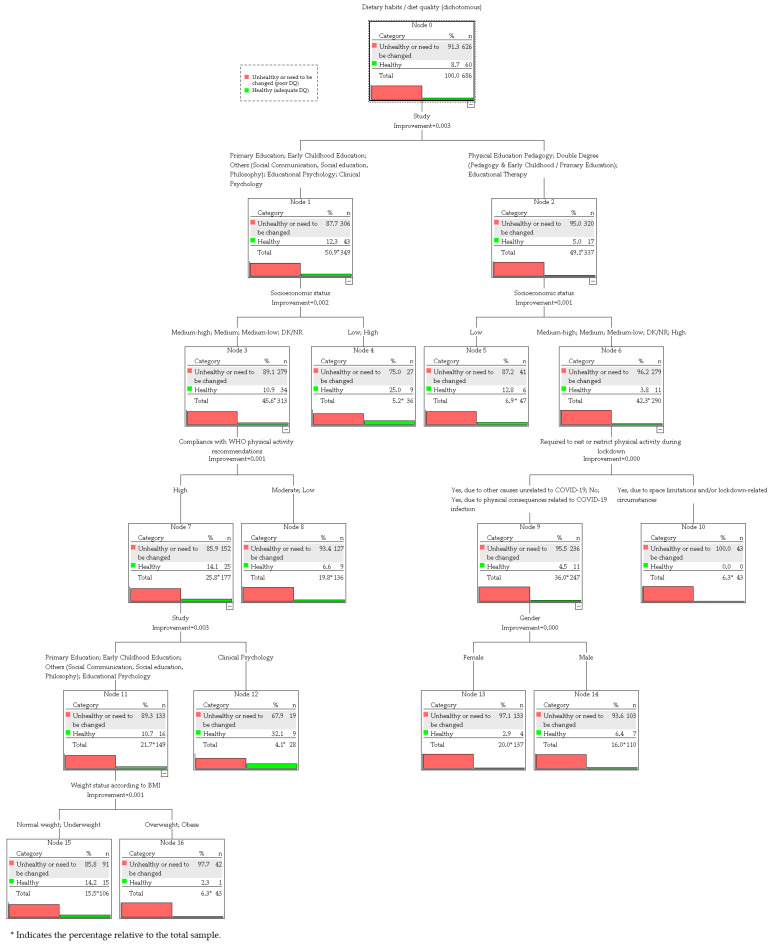
Classification and Regression Tree (CART) model for diet quality.

The CART analysis (Figure 2) identified eight predictors of diet quality (DQ), ordered according to their normalized variable importance as follows: field of study (100%), country (52.2%), socioeconomic status (SES, 48.9%), compliance with WHO physical activity (PA) recommendations (34.6%), body mass index (BMI, 23.4%), gender (20.1%), PA restriction during lockdown (15.7%), and academic year (8.2%).

Field of study was the strongest predictor (improvement = 0.007). Students enrolled in Physical Education Pedagogy, double-degree programs, Pedagogy, and Educational Therapy (Node 2) showed a 95.0% proportion of poor diet quality (DQ), compared with 87.7% among students in other majors (Node 1). Within Node 1, SES further stratified outcomes: intermediate SES students (Node 3) showed higher prevalence of poor DQ (89.1%) than low or high SES peers (75.0%, Node 4). WHO PA compliance further split Node 3, with non-compliant students (Node 8) reporting 93.4% poor DQ versus 85.9% in compliant peers (Node 7). Within Node 7, Clinical Psychology students (Node 12) showed a lower proportion of poor diet quality (67.9%) compared with students in other majors (Node 11, 89.3%). In this subgroup, BMI added discrimination: normal/underweight students (Node 15) had 85.8% poor DQ versus 97.7% in overweight/obese peers (Node 16).

For body-related majors (Node 2), socioeconomic status (SES) differentiated the risk: students with low SES (Node 5) had 87.2% poor diet quality (DQ), compared with 96.2% among those with higher SES (Node 6). Within Node 6, physical activity (PA) restriction during lockdown emerged as a significant predictor: all students whose PA was limited due to space constraints and/or lockdown-related circumstances showed poor diet quality (HEI ≤ 80). Finally, gender discriminated Node 9: female students (Node 13) reported 97.1% poor diets compared with 93.6% in males (Node 14).

The decision tree included five levels and 16 nodes, of which nine were terminal. The dependent variable exhibited a pronounced class imbalance, with 91.2% of students classified as having poor diet quality (HEI ≤ 80) and only 8.8% as healthy (HEI > 80). The model achieved an overall predictive accuracy of 91.3% (resubstitution error = 0.087, SE = 0.011); however, this performance was entirely driven by the majority class. The model showed zero sensitivity for the minority “healthy” category, reflecting its bias toward predicting the dominant class (i.e., poor DQ). The imbalance ratio between categories (poor vs. healthy DQ) was approximately 10:1 in both the CHAID and CART analyses, revealing a marked skew in class distribution that limited the discriminative capacity of both algorithms.

## 4. Discussion

This cross-national study enhances the understanding of diet quality among university students from Spain, Italy, Chile, and Mexico in the post-pandemic period. The findings reveal clear cross-country differences and distinct multidimensional risk profiles derived from both CHAID and CART segmentation analyses. Among all predictors, field of study showed the strongest association with Healthy Eating Index (HEI) scores, followed by country of residence. These associations suggest that both academic specialization and broader cultural and structural contexts may influence students’ dietary behaviors. Students from Italy and Spain exhibited comparatively favorable diet-quality profiles, whereas their peers in Chile and Mexico were disproportionately classified in the “poor” or “needs improvement” categories. These contrasts underscore how cultural traditions, socioeconomic realities, and environmental conditions intertwine in defining food choices [2,27].

Seen more broadly, our results indicate that nutritional inequalities reflect global shifts in eating habits. While European participants appeared to benefit from enduring elements of the Mediterranean model, students in Latin America were more exposed to the health risks associated with ultra-processed food consumption, sugar-sweetened beverages, and irregular eating schedules. If left unaddressed, such disparities could raise students’ cardiometabolic risk as they age. Therefore, these findings reinforce the need for context-sensitive public health strategies that account for local food environments, university infrastructures, and students’ lived experiences. Current evidence suggests that participatory approaches—particularly those that engage students in co-designing interventions, improving food literacy, and integrating experiential learning such as cooking labs—can substantially enhance adherence to healthy eating and food security among young adults [43,44].

Country-specific dietary habits help explain these gradients. Italian participants reported frequent consumption of cereals, vegetables, and fruit, a pattern aligned with the resilience of the Mediterranean diet and lifestyle among young Italians. This traditional model—characterized by plant-based foods, moderate dairy intake, and limited processed products—has been consistently linked to lower rates of depression, anxiety, and metabolic disorders, as well as enhanced life satisfaction and academic performance [45,46,47]. The persistence of these habits even among university students suggests that the Mediterranean-type dietary culture continues to serve as a protective behavioral and cultural framework that buffers against dietary deterioration.

Spanish students also displayed Mediterranean-oriented habits, though with notable differences. While their consumption of vegetables and fruits remained relatively high, the simultaneous increase in dairy, meat, fish, and processed products signals a gradual transformation toward more Westernized diets. Similar shifts have been documented in recent years, particularly during the COVID-19 lockdown, when mobility restrictions, emotional stress, and altered routines led to greater reliance on high-calorie, shelf-stable foods and takeout meals [29,30,31]. This subtle but persistent drift toward higher sugar and animal-fat intake highlights the tension between cultural continuity and modernization. Nonetheless, even under such transformations, Spanish and Italian students maintained overall better HEI scores, illustrating that the Mediterranean diet continues to exert a stabilizing effect on dietary quality across Southern Europe.

Recent reviews emphasize that the globalization of food markets and the widespread availability of ultra-processed foods have not fully displaced Mediterranean practices among young Europeans. Instead, a hybrid model is emerging, where traditional meal structures coexist with convenience-oriented behaviors [48]. This duality represents both a challenge and an opportunity: while it indicates the encroachment of less nutritious products, it also demonstrates that traditional frameworks retain influence and can be revitalized through targeted education and policy measures.

The dietary behaviors identified in Chile and Mexico stand in sharp contrast. Chilean students displayed persistently high consumption of sugar-sweetened beverages, consistent with national surveillance data showing that soda intake has remained elevated for nearly two decades despite successive public policies—such as front-of-pack warning labels and taxation on sugary drinks—aimed at curbing it [43]. This persistence reflects deep-rooted consumption habits reinforced by marketing practices and cultural acceptance of soft drinks as part of social and academic life.

Mexican students presented a more complex and ambivalent picture. On one hand, their dietary habits retain positive cultural elements, such as the frequent consumption of legumes—a staple with high fiber and micronutrient content that has been linked to improved metabolic outcomes and lower non-communicable disease risk [16]. On the other hand, this traditional strength is undermined by insufficient fruit and dairy intake, coupled with heavy consumption of industrial baked goods and sugary snacks rich in saturated fats. Early exposure to sweetened products, extensive marketing of processed foods, and socioeconomic inequalities appear to play key roles in shaping these habits [49].

Furthermore, evidence from Mexican university populations points to the influence of financial stress, food insecurity, and limited nutrition literacy as structural barriers to healthy eating [16]. The coexistence of cultural richness and economic vulnerability creates a paradox: while traditional Mexican diets are inherently balanced and nutrient-dense, economic and social pressures drive students toward cheap, energy-dense foods. The low HEI scores observed in Mexican participants confirm this double burden of cultural erosion and socioeconomic constraint. Addressing it requires interventions that go beyond individual behavior change to include affordability policies, campus-based access to healthy foods, and promotion of traditional yet sustainable eating habits.

Beyond geographical differences, academic and lifestyle factors substantially contributed to dietary outcomes. A striking result was that students enrolled in Physical Education Pedagogy—who might be expected to have greater awareness of health principles—reported poorer diet quality, whereas Psychology students tended to exhibit healthier eating patterns. This paradox is consistent with earlier studies showing that awareness of health topics often fails to translate into everyday healthy choices [43]. Possible explanations include time pressure from practical coursework, social eating dynamics, and a potential underestimation of nutrition compared with physical training.

This finding underscores the persistent gap between theoretical knowledge and behavioral implementation, pointing to the importance of embedding nutrition and food literacy into all academic disciplines, not only those directly related to health or sports. Evidence shows that integrative approaches—such as interdisciplinary modules or experiential learning combining theory with everyday practice—can enhance self-regulation and make healthy choices more habitual [50].

Physical activity (PA) levels further differentiated students’ dietary profiles. Those reporting lower PA or a reduction in activity compared with pre-pandemic levels consistently exhibited poorer diet quality. This pattern confirms the well-documented clustering of unhealthy behaviors, whereby physical inactivity, irregular sleep, and poor dietary habits often co-occur [6,17,47]. From a public health standpoint, the way these behaviors overlap helps explain why integrated programs are more effective than isolated efforts. Programs that simultaneously address nutrition, PA, and mental well-being—for example, through health-promoting campus initiatives—tend to yield more sustainable improvements in lifestyle behaviors.

Body weight also emerged as a meaningful correlate of dietary quality. Overweight and obese participants recorded significantly lower HEI scores, confirming the link between higher BMI and poorer diet quality in young adults [18]. This association reflects the energy imbalance driven by excess intake of processed foods, sugar-sweetened beverages, and refined carbohydrates, all of which contribute to increased adiposity and metabolic dysregulation [3,30].

University life itself constitutes a critical window for weight-related health risks. Multiple studies show that during the transition to adulthood, students face disruptions in routine, increased academic stress, and reduced parental oversight—factors that often lead to irregular eating habits, late-night meals, and sedentary habits [24]. Our data confirm that students of normal weight were significantly more likely to have higher diet-quality scores, reinforcing the argument that preventive measures targeting this population should be implemented before unhealthy weight trajectories are established. Such measures might include campus-based nutrition workshops, behavioral counseling, and digital interventions that promote healthy meal planning and time management.

The combined evidence suggests that overweight and obesity among university students are not merely outcomes of caloric excess but part of a broader web of interconnected behaviors—sedentary lifestyle, poor sleep, and psychosocial strain—[6,17,18,23] that require multifaceted institutional responses [6,10,13,45]. Integrating physical and mental wellness programs within university structures could be key to mitigating long-term cardiometabolic risks [3,5,18,45].

Socioeconomic status (SES) introduced another dimension of complexity. Among European students, higher SES was generally associated with better diet quality and healthier lifestyles, reflecting easier access to nutrient-rich foods and health-promoting environments [30,42,43]. Conversely, in Chile and Mexico, students from medium-to-high SES backgrounds paradoxically displayed lower diet quality. In these contexts, greater disposable income often correlates with higher consumption of fast food, convenience meals, and imported processed products [20].

This reversal of the usual socioeconomic pattern reflects the broader nutrition transition occurring in Latin America, where modernization and urbanization have diversified food availability but simultaneously increased exposure to low-cost, high-calorie options. Beyond economics, SES also interacts with psychosocial and behavioral factors such as stress, self-esteem, and sleep quality to shape dietary risks [16,34]. Consequently, interventions should consider not only affordability and availability but also psychological and cultural drivers of consumption, recognizing that social aspirations and marketing pressures can powerfully influence young adults’ eating behaviors.

A key strength of this study is the combined use of two decision-tree models (Exhaustive CHAID and CART), which allowed us to explore how different variables interact in relation to diet quality. The rationale for this combined approach is both conceptual and methodological. CHAID identifies statistically significant multiway splits, revealing hierarchical relationships among categorical variables, whereas CART generates binary splits that optimize predictive accuracy through pruning and cross-validation [19].

By integrating both, the analyses achieved a balance between explanatory transparency and predictive precision. CHAID highlighted structural correlates of diet quality—such as country, SES, and living arrangements—while CART identified more proximal behavioral and demographic factors including PA level, BMI, academic field, and gender. Because both models identified similar predictors, the resulting profiles can be considered robust and consistent. Nevertheless, both algorithms were affected by a pronounced class imbalance between categories of diet quality (poor vs. healthy ≈ 10:1), which limited their discriminative capacity despite high overall accuracy. This methodological constraint should be considered when interpreting the predictive strength of the models and reinforces the need for balanced or weighted approaches in future analyses.

Moreover, this methodological synergy aligns with recent advances in nutritional epidemiology advocating for data-driven segmentation methods to complement regression-based approaches [27,28].

When compared with international studies, our results reveal comparable dietary risk profiles among university populations, ranging from healthier to less healthy clusters, yet the present decision-tree approach provides clearer and more interpretable categories. Similar to the behavioral clustering analyses conducted in Ireland [23] and in Australia [51], our models indicate that diet quality among students is not dichotomous but unfolds along a continuum shaped by sociodemographic, academic, and lifestyle dimensions. Likewise, studies in the United Kingdom [52], Finland [53], and Southeast Asia [54] have described parallel co-occurrence patterns linking poor dietary habits, sedentary behavior, and psychosocial distress. In contrast to traditional cluster or regression-based methods, the hierarchical structure of the CHAID and CART algorithms used here allowed for a stepwise visualization of how contextual and behavioral variables interact at successive levels to explain variations in diet quality. This predictive, hierarchy-based perspective complements previous international evidence by revealing not only who is more likely to exhibit poor diet quality but also how combinations of academic, socioeconomic, and lifestyle factors are associated with that outcome.

The study’s findings have direct practical implications. In European settings, maintaining the resilience of Mediterranean practices is essential while mitigating the progressive adoption of Westernized patterns. Universities can serve as critical agents in this process by regulating food environments, limiting the availability of ultra-processed items, and incorporating healthy defaults in canteens and vending facilities.

In Latin American contexts, structural interventions are equally urgent. Improving campus food offerings, expanding access to affordable fresh produce, taxing sugar-sweetened beverages, and promoting legume-based traditional dishes could contribute to reversing current unfavorable trends. At the institutional level, the behavioral determinants identified through CART underscore the need for integrated campus strategies that combine nutrition education, opportunities for PA, and psychosocial support, aligned with the principles of the updated “health-promoting campus” model [34].

Several limitations must be acknowledged. The non-probability sampling approach restricts representativeness and limits generalization to the broader university population. The cross-sectional design prevents causal inference, leaving open the directionality between diet quality and associated factors such as PA or BMI. Additionally, reliance on self-reported data introduces potential recall and desirability biases, and although the HEI is validated internationally, it may not capture culturally specific dietary nuances across four distinct countries. Class imbalance within the predictive models could have reduced sensitivity to identifying students with optimal diets, potentially underestimating positive cases. Finally, while frequency data allowed meaningful cross-country comparisons of diet quality, the study did not include information on portion size, nutrient intake, or meal timing; therefore, references to patterns should be understood in a behavioral—not compositional—sense.

Despite these constraints, this study offers a valuable empirical and methodological contribution. The cross-national design allows for meaningful cultural comparisons, and the application of dual decision-tree models provides a robust and interpretable framework for identifying both structural and behavioral predictors of poor diet quality.

Looking ahead, future research should adopt longitudinal and interventional designs to determine the causal sequence between diet, PA, and psychosocial factors. Incorporating biomarkers such as lipid profiles, inflammatory markers, and gut microbiota composition could also help link dietary habits to objective health outcomes. Moreover, extending similar analyses to other world regions—such as North America, Asia, and Africa—would enrich understanding of how global and local forces interact in shaping young adults’ diets in an era of rapid sociocultural transformation.

## 5. Conclusions

This multicountry study demonstrates that university students’ diet quality results from the interplay of structural (cultural and socioeconomic) and proximal (academic and behavioral) factors. Using two complementary segmentation algorithms (CHAID and CART), we identified non-linear interactions in which country of residence and socioeconomic status—together with field of study, physical activity, weight status, and gender—emerged as the most influential predictors of poor diet quality. These results confirm that students’ nutritional behaviors arise from multidimensional constellations shaped by context and lifestyle rather than from isolated risk factors.

The findings emphasize the need for coordinated strategies at the personal, institutional, and policy levels to improve diet quality among university students. In European settings, preserving adherence to Mediterranean dietary patterns remains a public health priority, reinforcing cultural resilience while counteracting the gradual adoption of Westernized habits. In Latin American universities, efforts should focus on reducing structural inequalities in food access and affordability and on improving campus food environments. Reviving local dietary practices—particularly those centered on legumes and minimally processed foods—may represent a culturally grounded route toward nutritional sustainability.

Across all contexts, integrating nutrition education, opportunities for physical activity, and psychosocial well-being programs within university infrastructures is consistent with the Okanagan Charter and the Health-Promoting University framework. Such an integrated approach goes beyond awareness campaigns, fostering environments where healthy choices become easy, affordable, and habitual.

Although the non-probability sampling, cross-sectional design, and self-reported data limit causal inference, the dual-decision-tree model enhances interpretability and provides exploratory insight into complex behavioral profiles. The predictive performance observed supports the utility of machine learning segmentation for identifying subgroups at dietary risk in young adult populations.

Future research should adopt longitudinal and intervention designs incorporating objective biomarkers and mental health measures to better capture the bidirectional links between diet, physical health, and psychological well-being. Ultimately, improving diet quality in higher education settings represents not only a nutritional goal but also a key pathway toward greater equity and comprehensive student well-being.

## Figures and Tables

**Table 1:** Sociodemographic characteristics (categorical variables) of university students by country of origin.

		Total (n = 686)	Chile (n = 161)	Spain (n = 180)	Italy (n = 183)	Mexico (n = 162)
Variables	Groups	*f*_i_(%)	*f*_i_(%)[R] †	*f*_i_(%)[R] †	*f*_i_(%)[R] †	*f*_i_(%)[R] †
Gender ***	Female	417(60.8)	57(35.4)[−7.5]	109(60.6)[−0.1]	134(73.2)[4.0]	117(72.2)[3.4]
	Male	269(39.2)	104(64.6)[7.5]	71(39.4)[0.1]	49(26.8)[−4.0]	45(27.8)[−3.4]
Study ***	Pedagogy	129(18.8)	0(0.0)[−7.0]	27(15.0)[−1.5]	1(0.5)[−7.4]	101(62.3)[16.2]
	Early Childhood Education	15(2.2)	0(0.0)[−2.2]	15(8.3)[6.6]	0(0.0)[−2.4]	0(0.0)[−2.2]
	Primary Education	130(19.0)	0(0.0)[−7.0]	130(72.2)[21.2]	0(0.0)[−7.6]	0(0.0)[−7.0]
	Physical Education Pedagogy	161(23.5)	161(100)[26.2]	0(0.0)[−8.7]	0(0.0)[−8.7]	0(0.0)[−8.1]
	Clinical Psychology	67(9.8)	0(0.0)[−4.8]	0(0.0)[−5.1]	67(36.6)[14.3]	0(0.0)[−4.8]
	Educational Psychology	88(12.8)	0(0.0)[−5.6]	0(0.0)[−6.0]	74(40.4)[13.0]	14(8.6)[−1.8]
	Educational Therapy	47(6.9)	0(0.0)[−3.9]	0(0.0)[−4.2]	0(0.0)[−4.3]	47(29.0)[12.8]
	Social and Vocational Pedagogies and Others	49(7.1)	0(0.0)[−4.0]	8(4.4)[−1.6]	41(22.4)[9.4]	0(0.0)[−4.0]
Year ***	First	245(35.7)	75(46.6)[3.3]	62(34.4)[−0.4]	36(19.7)[−5.3]	72(44.4)[2.7]
	Second	94(13.7)	4(2.5)[−4.7]	59(32.8)[8.7]	16(8.7)[−2.3]	15(9.3)[−1.9]
	Third	115(16.8)	22(13.7)[−1.2]	22(12.2)[−1.9]	16(8.7)[−3.4]	55(34.0)[6.7]
	Fourth	105(15.3)	8(5.0)[−4.2]	37(20.6)[2.3]	52(28.4)[5.8]	8(4.9)[−4.2]
	Fifth	127(18.5)	52(32.3)[5.1]	0(0.0)[−7.4]	63(34.4)[6.5]	12(7.4)[−4.2]
Domestic cohabitation ***	With immediate family members	575(83.8)	130(80.7)[−1.2]	165(91.7)[3.3]	131(71.6)[−5.2]	149(92.0)[3.2]
	With non-family members	111(16.2)	31(19.3)[1.2]	15(8.3)[−3.3]	52(28.4)[5.2]	13(8.0)[−3.2]
Number of cohabitants ***	One (living alone)	28(4.1)	9(5.6)[1.1]	7(3.9)[−0.2]	3(1.6)[−1.9]	9(5.6)[1.1]
	Two	87(12.7)	18(11.2)[−0.7]	21(11.7)[−0.5]	34(18.6)[2.8]	14(8.6)[−1.8]
	Three	155(22.6)	40(24.8)[0.8]	36(20.0)[−1.0]	48(26.2)[1.4]	31(19.1)[−1.2]
	Four	231(33.7)	44(27.3)[−1.9]	91(50.6)[5.6]	47(25.7)[−2.7]	49(30.2)[−1.1]
	Five or more	185(27.0)	50(31.1)[1.3]	25(13.9)[−4.6]	51(27.9)[0.3]	59(36.4)[3.1]
Socioeconomic level ***	High	34(5.0)	1(0.6)[−2.9]	27(15.0)[7.2]	5(2.7)[−1.6]	1(0.6)[−2.9]
	Higher-middle	100(14.6)	9(5.6)[−3.7]	43(23.9)[4.1]	48(26.2)[5.2]	0(0.0)[−6.0]
	Middle	337(49.1)	50(31.1)[−5.2]	85(47.2)[−0.6]	79(43.2)[−1.9]	123(75.9)[7.8]
	Lower-middle	99(14.4)	61(37.9)[9.7]	21(11.7)[−1.2]	17(9.3)[−2.3]	0(0.0)[−6.0]
	Low	55(8.0)	27(16.8)[4.7]	2(1.1)[−4.0]	4(2.2)[−3.4]	22(13.6)[3.0]
	Don’t know/No response	61(8.9)	13(8.1)[−0.4]	2(1.1)[−4.3]	30(16.4)[4.2]	16(9.9)[0.5]
Employment status ***	Employed	263(38.3)	60(37.3)[−0.3]	98(54.4)[5.2]	71(38.8)[0.1]	34(21.0)[−5.2]
	Unemployed	423(61.7)	101(62.7)[0.3]	82(45.6)[−5.2]	112(61.2)[−0.1]	128(79.0)[5.2]
Financial status *	Fully dependent	406(59.2)	95(59.0)[−0.1]	97(53.9)[−1.7]	103(56.3)[−0.9]	111(68.5)[2.8]
	Covers <50% of expenses	153(22.3)	26(16.1)[−2.1]	54(30.0)[2.9]	45(24.6)[0.9]	28(17.3)[−1.8]
	Covers >50% of expenses	81(11.8)	27(16.8)[2.2]	21(11.7)[−0.1]	21(11.5)[−0.2]	12(7.4)[−2.0]
	Fully independent	46(6.7)	13(8.1)[0.8]	8(4.4)[−1.4]	14(7.7)[0.6]	11(6.8)[0.0]
Under treatment ***	Medical	58(8.5)	13(8.1)[−0.2]	18(10.0)[0.9]	20(10.9)[1.4]	7(4.3)[−2.2]
	Psychological	72(10.5)	12(7.5)[−1.4]	11(6.1)[−2.2]	39(21.3)[5.6]	10(6.2)[−2.1]
	No	556(81.0)	136(84.5)[1.3]	151(83.9)[1.1]	124(67.8)[−5.4]	145(89.5)[3.1]
On a special diet *	Yes	51(7.4)	8(5.0)[−1.4]	15(8.3)[0.5]	22(12.0)[2.8]	6(3.7)[−2.1]
	No	635(92.6)	153(95.0)[1.4]	165(91.7)[−0.5]	161(88.0)[−2.8]	156(96.3)[2.1]
Previously infected with COVID-19 ***	Not to my knowledge	345(50.3)	107(66.5)[4.7]	62(34.4)[−5.0]	70(38.3)[−3.8]	106(65.4)[4.4]
	Based on self-administered test results	130(19.0)	11(6.8)[−4.5]	52(28.9)[4.0]	43(23.5)[1.8]	24(14.8)[−1.5]
	Diagnosed by a physician	211(30.8)	43(26.7)[−1.3]	66(36.7)[2.0]	70(38.3)[2.6]	32(19.8)[−3.5]
Rest or restriction of physical activity during COVID-19 lockdown ***	No	273(39.8)	55(34.2)[−1.7]	77(42.8)[1.0]	64(35.0)[−1.6]	77(47.5)[2.3]
	Associated with infection	110(16.0)	24(14.9)[−0.4]	17(9.4)[−2.8]	41(22.4)[2.7]	28(17.3)[0.5]
	Unrelated to COVID-19	211(30.8)	72(44.7)[4.4]	80(44.4)[4.6]	50(27.3)[−1.2]	9(5.6)[−8.0]
	Space limitations/lockdown circumstances	92(13.4)	10(6.2)[−3.1]	6(3.3)[−4.6]	28(15.3)[0.9]	48(29.6)[6.9]
Pre- and post-pandemic physical activity level ***	Much more PA now than before the pandemic	141(20.6)	68(42.2)[7.8]	28(15.6)[−1.9]	25(13.7)[−2.7]	20(12.3)[−3.0]
	More PA now	149(21.7)	37(23.0)[0.4]	39(21.7)[0.0]	30(16.4)[−2.0]	43(26.5)[1.7]
	Similar PA pre- and post-pandemic	208(30.3)	28(17.4)[−4.1]	58(32.2)[0.6]	74(40.4)[3.5]	48(29.6)[−0.2]
	Less PA now	140(20.4)	24(14.9)[−2.0]	39(21.7)[0.5]	37(20.2)[−0.1]	40(24.7)[1.5]
	Much less PA in the post-pandemic period	48(7.0)	4(2.5)[−2.6]	16(8.9)[1.2]	17(9.3)[1.4]	11(6.8)[−0.1]
Habitual physical activity level ***	High	407(59.3)	123(76.4)[5.0]	112(62.2)[0.9]	92(50.3)[−2.9]	80(49.4)[−2.9]
	Moderate	166(24.2)	30(18.6)[−1.9]	50(27.8)[1.3]	35(19.1)[−1.9]	51(31.5)[2.5]
	Low	113(16.5)	8(5.0)[−4.5]	18(10.0)[−2.7]	56(30.6)[6.0]	31(19.1)[1.0]
BMI-based weight status *	Underweight	46(6.7)	4(2.5)[−2.4]	10(5.6)[−0.7]	22(12.0)[3.4]	10(6.2)[−0.3]
	Normal weight	434(63.3)	102(63.4)[0.0]	130(72.2)[2.9]	117(63.9)[0.2]	85(52.5)[−3.3]
	Overweight	168(24.5)	45(28.0)[1.2]	37(20.6)[−1.4]	35(19.1)[−2.0]	51(31.5)[2.4]
	Obese	38(5.5)	10(6.2)[0.4]	3(1.7)[−2.6]	9(4.9)[−0.4]	16(9.9)[2.8]

* *p* < 0.05/*** *p*< 0.001 χ^2^ test (variable × country). † *f*_i_(%)[R]: Frequency (Percentage) and Adjusted standardized residuals.

**Table 2:** Sociodemographic characteristics (continuous variables) of university students by country of origin.

	Total		Chile		Spain		Italy		Mexico	
Variables	N	Mean ± SD	N	Mean ± SD	N	Mean ± SD	N	Mean ± SD	N	Mean ± SD
Age ***	685	22.41 ± 5.05	160	21.62 ± 2.85	180	20.96 ± 2.18	183	26.27 ± 7.62	162	20.43 ± 2.41
Hours worked per week ***	365	8.93 ± 12.15	51	14.60 ± 9.36	90	12.15 ± 8.39	62	13.17 ± 16.12	162	3.73 ± 10.93
METs-min/week spent on VPA ***	686	1970.33 ± 2325.86	161	2619.03 ± 2499.21	180	1736.27 ± 1963.43	183	1951.26 ± 2345.97	162	1607.23 ± 2384.60
METs-min/week spent on MPA ***	686	790.57 ± 1008.50	161	1040.99 ± 1061.06	180	502.13 ± 618.45	183	830.27 ± 1142.74	162	817.31 ± 1071.82
METs-min/week spent on walking **	686	1419.44 ± 1276.13	161	1649.51 ± 1363.66	180	1208.22 ± 1107.06	183	1639.36 ± 1458.65	162	1177.05 ± 1046.92
Total METs-min/week ***	686	4180.37 ± 3412.10	161	5309.56 ± 3784.12	180	3446.70 ± 2491.52	183	4420.89 ± 3631.06	162	3601.64 ± 3349.35
Body Mass Index ***	686	23.48 ± 6.70	161	24.16 ± 3.41	180	22.41 ± 3.17	183	22.43 ± 4.29	162	25.16 ± 11.90

** *p* < 0.01/*** *p* < 0.001 (variable × country).

**Table 3:** Frequency of food consumption in the total sample.

Food Group	Group	Never or Almost Never *f*_i_(%)[R] †	Once a Week *f*_i_(%)[R] †	1–2 Times a Week *f*_i_(%)[R] †	≥3 Times a Week *f*_i_(%)[R] †	Every Day *f*_i_(%)[R] †
Cereals ***	Total	22(3.2)	10(1.5)	64(9.3)	198(28.9)	392(57.1)
	Chile	3(1.9)[−1.1]	4(2.5)[1.2]	14(8.7)[−0.3]	41(25.5)[−1.1]	99(61.5)[1.3]
	Spain	4(2.2)[−0.9]	4(2.2)[1.0]	14(7.8)[−0.8]	61(33.9)[1.7]	97(53.9)[−1.0]
	Italy	9(4.9)[1.5]	2(1.1)[−0.5]	14(7.7)[−0.9]	31(16.9)[−4.2]	127(69.4)[3.9]
	Mexico	6(3.7)[0.4]	0(0.0)[−1.8]	22(13.6)[2.1]	65(40.1)[3.6]	69(42.6)[−4.3]
Vegetables ***	Total	13(1.9)	18(2.6)	104(15.2)	228(33.2)	323(47.1)
	Chile	3(1.9)[0.0]	5(3.1)[0.4]	30(18.6)[1.4]	41(25.5)[−2.4]	82(50.9)[1.1]
	Spain	4(2.2)[0.4]	9(5.0)[2.3]	43(23.9)[3.8]	56(31.1)[−0.7]	68(37.8)[−2.9]
	Italy	4(2.2)[0.3]	4(2.2)[−0.4]	14(7.7)[−3.3]	44(24.0)[−3.1]	117(63.9)[5.3]
	Mexico	2(1.2)[−0.7]	0(0.0)[−2.4]	17(10.5)[−1.9]	87(53.7)[6.3]	56(34.6)[−3.7]
Fruits ***	Total	48(7.0)	46(6.7)	135(19.7)	199(29.0)	258(37.6)
	Chile	9(5.6)[−0.8]	14(8.7)[1.2]	42(26.1)[2.3]	39(24.2)[−1.5]	57(35.4)[−0.7]
	Spain	19(10.6)[2.2]	19(10.6)[2.4]	33(18.3)[−0.5]	37(20.6)[−2.9]	72(40.0)[0.8]
	Italy	18(9.8)[1.8]	13(7.1)[0.3]	31(16.9)[−1.1]	46(25.1)[−1.3]	75(41.0)[1.1]
	Mexico	2(1.2)[−3.3]	0(0.0)[−3.9]	29(17.9)[−0.7]	77(47.5)[5.9]	54(33.3)[−1.3]
Dairy products ***	Total	25(3.6)	21(3.1)	117(17.1)	209(30.5)	314(45.8)
	Chile	4(2.5)[−0.9]	8(5.0)[1.6]	17(10.6)[−2.5]	59(36.6)[1.9]	73(45.3)[−0.1]
	Spain	1(0.6)[−2.6]	3(1.7)[−1.3]	21(11.7)[−2.2]	24(13.3)[−5.8]	131(72.8)[8.5]
	Italy	11(6.0)[2.0]	10(5.5)[2.2]	36(19.7)[1.1]	49(26.8)[−1.3]	77(42.1)[−1.2]
	Mexico	9(5.6)[1.5]	0(0.0)[−2.6]	43(26.5)[3.7]	77(47.5)[5.4]	33(20.4)[−7.4]
Meat, fish, or eggs ***	Total	21(3.1)	10(1.5)	103(15.0)	289(42.1)	263(38.3)
	Chile	9(5.6)[2.1]	5(3.1)[2.0]	18(11.2)[−1.6]	62(38.5)[−1.1]	67(41.6)[1.0]
	Spain	2(1.1)[−1.8]	1(0.6)[−1.2]	20(11.1)[−1.7]	72(40.0)[−0.7]	85(47.2)[2.9]
	Italy	4(2.2)[−0.8]	4(2.2)[1.0]	28(15.3)[0.1]	71(38.8)[−1.1]	76(41.5)[1.0]
	Mexico	6(3.7)[0.5]	0(0.0)[−1.8]	37(22.8)[3.2]	84(51.9)[2.9]	35(21.6)[−5.0]
Legumes ***	Total	69(10.1)	108(15.7)	273(39.8)	193(28.1)	43(6.3)
	Chile	18(11.2)[0.5]	45(28.0)[4.9]	59(36.6)[−0.9]	33(20.5)[−2.5]	6(3.7)[−1.5]
	Spain	11(6.1)[−2.1]	25(13.9)[−0.8]	97(53.9)[4.5]	37(20.6)[−2.6]	10(5.6)[−0.5]
	Italy	31(16.9)[3.6]	38(20.8)[2.2]	65(35.5)[−1.4]	35(19.1)[−3.2]	14(7.7)[0.9]
	Mexico	9(5.6)[−2.2]	0(0.0)[−6.3]	52(32.1)[−2.3]	88(54.3)[8.5]	13(8.0)[1.1]
Processed meats ***	Total	133(19.4)	98(14.3)	239(34.8)	159(23.2)	57(8.3)
	Chile	36(22.4)[1.1]	25(15.5)[0.5]	56(34.8)[0.0]	34(21.1)[−0.7]	10(6.2)[−1.1]
	Spain	19(10.6)[−3.5]	28(15.6)[0.6]	58(32.2)[−0.9]	41(22.8)[−0.1]	34(18.9)[6.0]
	Italy	53(29.0)[3.8]	45(24.6)[4.7]	51(27.9)[−2.3]	26(14.2)[−3.4]	8(4.4)[−2.3]
	Mexico	25(15.4)[−1.5]	0(0.0)[−5.9]	74(45.7)[3.3]	58(35.8)[4.4]	5(3.1)[−2.8]
Sweets and commercially baked goods (pastries, treats, candies) ***	Total	118(17.2)	98(14.3)	185(27.0)	198(28.9)	87(12.7)
	Chile	19(11.8)[−2.1]	23(14.3)[0.0]	50(31.1)[1.3]	48(29.8)[0.3]	21(13.0)[0.2]
	Spain	49(27.2)[4.1]	32(17.8)[1.6]	49(27.2)[0.1]	36(20.0)[−3.1]	14(7.8)[−2.3]
	Italy	30(16.4)[−0.3]	43(23.5)[4.2]	37(20.2)[−2.4]	43(23.5)[−1.9]	30(16.4)[1.8]
	Mexico	20(12.3)[−1.9]	0(0.0)[−5.9]	49(30.2)[1.1]	71(43.8)[4.8]	22(13.6)[0.4]
Sugar-sweetened beverages ***	Total	206(30.0)	94(13.7)	190(27.7)	121(17.6)	75(10.9)
	Chile	26(16.1)[−4.4]	15(9.3)[−1.8]	31(19.3)[−2.7]	48(29.8)[4.6]	41(25.5)[6.8]
	Spain	69(38.3)[2.8]	37(20.6)[3.1]	46(25.6)[−0.7]	17(9.4)[−3.4]	11(6.1)[−2.4]
	Italy	80(43.7)[4.7]	42(23.0)[4.2]	38(20.8)[−2.4]	16(8.7)[−3.7]	7(3.8)[−3.6]
	Mexico	31(19.1)[−3.5]	0(0.0)[−5.8]	75(46.3)[6.1]	40(24.7)[2.7]	16(9.9)[−0.5]

*** *p*< 0.001 χ^2^ test (variable × country). † *f*_i_(%)[R]: Frequency (Percentage) and Adjusted standardized residuals.

**Table 4:** Prevalence of dietary habit categories according to the Healthy Eating Index (HEI) across categorical variables showing statistically significant differences.

Variable	Group	Unhealthy *f*_i_(%)[R] †	Need Modifications *f*_i_(%)[R] †	Healthy *f*_i_(%)[R] †
	Total	96(14.0)	530(77.3)	60(8.7)
Country ***	Chile	30(18.6)[1.9]	121(75.2)[−0.7]	10(6.2)[−1.3]
	Spain	16(8.9)[−2.3]	146(81.1)[1.4]	18(10.0)[0.7]
	Italy	17(9.3)[−2.1]	140(76.5)[−0.3]	26(14.2)[3.1]
	Mexico	33(20.4)[2.7]	123(75.9)[−0.5]	6(3.7)[−2.6]
Study *	Pedagogy	28(21.7)[2.8]	96(74.4)[−0.9]	5(3.9)[−2.2]
	Early Childhood Education	2(13.3)[−0.1]	11(73.3)[−0.4]	2(13.3)[0.6]
	Primary Education	9(6.9)[−2.6]	107(82.3)[1.5]	14(10.8)[0.9]
	Physical Education Pedagogy	30(18.6)[2.7]	121(75.2)[−0.7]	10(6.2)[−1.3]
	Clinical Psychology	7(10.4)[−0.9]	49(73.1)[−0.8]	11(16.4)[2.3]
	Educational Psychology	8(9.1)[−1.4]	70(79.5)[0.5]	10(11.4)[0.9]
	Educational Therapy	6(12.8)[−0.3]	39(83.0)[1.0]	2(4.3)[−1.1]
	Social and Vocational Pedagogies. and Others	6(12.2)[−0.4]	37(75.5)[−0.3]	6(12.2)[0.9]
Year *	First	39(15.9)[1.1]	190(77.6)[0.1]	16(6.5)[−1.5]
	Second	9(9.6)[−1.3]	74(78.7)[0.4]	11(11.7)[1.1]
	Third	19(16.5)[0.9]	88(76.5)[−0.2]	8(7.0)[−0.7]
	Fourth	8(7.6)[−2.0]	80(76.2)[−0.3]	17(16.2)[2.9]
	Fifth	21(16.5)[0.9]	98(77.2)[0.0]	8(6.3)[−1.1]
Habitual physical activity level *	High	54(13.3)[−0.7]	310(76.2)[−0.8]	43(10.6)[2.0]
	Moderate	17(10.2)[−1.6]	136(81.9)[1.6]	13(7.8)[−0.5]
	Low	25(22.1)[2.7]	84(74.3)[−0.8]	4(3.5)[−2.1]

* *p*< 0.05/*** *p*< 0.001 χ^2^ test (variable × country). † *f*_i_(%)[R]: Frequency (Percentage) [Adjusted standardized residuals].

**Table 5:** HEI by variables showing statistically significant differences.

Variable	Groups	Min.	Máx.	Mean ± SD
	Total	24.5	100	63.71 ± 12.66
Country ***	Chile	32.0	93.0	61.86 ± 12.55
	Spain	27.5	89.5	66.38 ± 11.14
	Italy	33.0	100	67.08 ± 12.65
	Mexico	24.5	88.0	58.77 ± 12.55
Study ***	Pedagogy	32.5	88.0	59.29 ± 12.28
	Early Childhood Education	42.5	87.5	66.93 ± 12.04
	Primary Education	27.5	89.5	66.93 ± 11.02
	Physical Education Pedagogy	32.0	93.0	61.86 ± 12.55
	Clinical Psychology	39.0	94.0	66.83 ± 13.28
	Educational Psychology	24.5	100	67.23 ± 13.07
	Educational Therapy	35.0	87.0	59.28 ± 11.66
	Social and Vocational Pedagogies. and Others	33.0	85.5	65.55 ± 12.42
Year ***	First	27.5	93.0	62.0 ± 12.54
	Second	41.0	89.5	66.31 ± 11.13
	Third	32.5	100	61.80 ± 13.17
	Fourth	24.5	94.0	67.94 ± 12.52
	Fifth	32.0	89.5	63.31 ± 12.63
Socioeconomic level ***	High	53.0	93.0	72.10 ± 9.66
	Higher-middle	33.0	94.0	64.71 ± 12.02
	Middle	24.5	92.0	62.97 ± 13.07
	Lower-middle	37.5	87.0	63.71 ± 11.07
	Low	32.0	93.0	60.71 ± 13.51
	Don’t know/No response	35.0	100	64.17 ± 12.89
Number of cohabitants *	One (living alone)	24.5	81.0	57.00 ± 16.47
	Two	33.0	94.0	66.33 ± 12.70
	Three	32.5	89.5	64.40 ± 11.69
	Four	32.5	93.0	62.60 ± 12.15
	Five or more cohabitants	27.5	100	64.30 ± 13.03
Pre- and post-pandemic physical activity level ***	Much more PA now than before the pandemic	24.5	93.0	64.01 ± 12.66
	More PA now	32.0	94.0	66.34 ± 12.04
	Similar PA pre- and post-pandemic	27.5	100	64.25 ± 12.59
	Less PA now	29.0	87.5	60.16 ± 12.71
	Much less PA in the post-pandemic period	32.5	88.0	62.66 ± 12.80
Habitual physical activity level *	High	24.5	100	64.41 ± 13.30
	Moderate	33.0	87.5	63.70 ± 11.56
	Low	37.5	89.5	61.20 ± 11.56
BMI-based weight status *	Underweight	35.0	89.5	64.72 ± 12.11
	Normal weight	27.5	100	64.11 ± 12.26
	Overweight	29.0	93.0	63.75 ± 13.01
	Obese	24.5	86.5	57.76 ± 15.04

* *p* < 0.05/*** *p* < 0.001.

## Data Availability

All data supporting the findings of this study are contained within the article. Additional information can be provided by the corresponding author upon reasonable request.

## Abbreviations

The following abbreviations are used in this manuscript:

COVID-19	Coronavirus Disease 2019
IPAQ-SF	International Physical Activity Questionnaire—Short Form
HEI	Healthy Eating Index
ANOVA	Analysis of Variance
CHAID	Chi-squared Automatic Interaction Detection
CART	Classification and Regression Trees
WHO	World Health Organization
SES	Socioeconomic Status
PA	Physical Activity
ICAR	Research Committee for the Attention to Research
BMI	Body Mass Index
